# Axillary Nerve Palsy Following Latissimus Dorsi Tendon Transfer for Irreparable Rotator Cuff Tears

**DOI:** 10.7759/cureus.107532

**Published:** 2026-04-22

**Authors:** Mikel Aramberri-Gutiérrez, Giovanni Tiso-D Orazio, Gustavo E Dávila-Godínez, Iñaki Mediavilla, Enrico Gervasi

**Affiliations:** 1 Sports Medicine, ALAI Sports Medicine Clinic, Madrid, ESP; 2 Orthopedics, Hospital Español de México, Mexico City, MEX; 3 Sports Medicine, Hospital Universitario de Basurto, Bilbao, ESP; 4 Shoulder Surgery, Latisana Civil Hospital, Latisana, ITA

**Keywords:** axillary nerve neuropraxia, latissimus dorsi, massive rotator cuff tear, rotator cuff retear, tendon transfer

## Abstract

Recurrent rotator cuff tears in young patients represent a complex therapeutic challenge. Latissimus dorsi tendon transfers (LDTT) are considered an effective treatment option for massive tears with deficiencies of the posterosuperior rotator cuff. However, the procedure can still produce complications, among which neurological complications are rare, particularly those involving the axillary nerve. This review presents the case of a 53-year-old male patient with an extensive, irreparable posterosuperior rotator cuff retear who underwent arthroscopically assisted LDTT without intraoperative complications. The initial postoperative course was as expected, with minimal pain and gradual improvement in passive range of motion. However, at seven weeks, delayed progression of mobility was noted, associated with hypoesthesia in the axillary nerve distribution and deltoid hypotrophy. This prompted an electrodiagnostic study. Electromyography (EMG) revealed severe axillary nerve neuropraxia with signs of acute axonal degeneration. Management was conservative, with intensive rehabilitation and close clinical follow-up. By 14 months of follow-up, the patient achieved full recovery of mobility, strength, and sensibility, with both clinical and EMG resolution of the neuropraxia.

## Introduction

Retear of the rotator cuff, particularly in young patients, represents a therapeutic challenge even for the most experienced shoulder surgeons. It is one of the main complications following either arthroscopic or open repairs, with a reported prevalence ranging from 13% to 94% of cases [1]. Described postoperative risk factors include age, characteristics of the lesion, fatty degeneration, and suture technique [2]. After retear, patients present in the clinic with pain, weakness, stiffness, and loss of postoperative functional progress [3]. 

Magnetic resonance imaging (MRI) is the imaging study used to identify these cases. Its diagnostic accuracy for detecting retears or failed repairs ranges from 70% to 90%, improving when intra-articular contrast is used [3]. Currently, several therapeutic options are available, depending on the characteristics of the lesion, as well as the patient’s symptoms and functional status. Tendon transfer is a surgical option for young and functionally active patients with poor quality or insufficient length in their remaining rotator cuff tendons [3]. Its primary goal is to restore joint biomechanics, thus improving function and relieving pain [4]. 

In posterosuperior rotator cuff tears, the latissimus dorsi (LD) is the most commonly used tendon, according to the technique introduced by Gerber et al. in 1988 [5]. This technique consists of reattaching the tendon from the anteromedial humeral neck to the anterolateral region of the greater tuberosity of the humerus. This redirection modifies the force vector, transforming its primary action from internal rotation and extension to external rotation and shoulder flexion. [6]. This biomechanical rebalancing between internal and external rotation forces helps restore the glenohumeral fulcrum, allowing the deltoid muscle to act more efficiently as the main elevator of the shoulder [7]. 

Arthroscopically assisted approaches are an alternative that provides clinical outcomes similar to or even superior to open approaches, while also offering lower surgical morbidity [6,8]. Reported complication rates range from 7.3% to 18.9%, with tendon retear being the most frequent, followed by infection and hematoma; neurological injury is rare and usually involves isolated cases of radial nerve involvement [6,8]. 

## Case presentation

A 53-year-old male patient with high functional physical activity demands presented with a history of arthroscopic rotator cuff repair following a massive traumatic tear one year prior to evaluation at our center. He reported persistent and progressive pain since the postoperative period, with no improvement despite rehabilitation treatment. 

**Figure 1:**
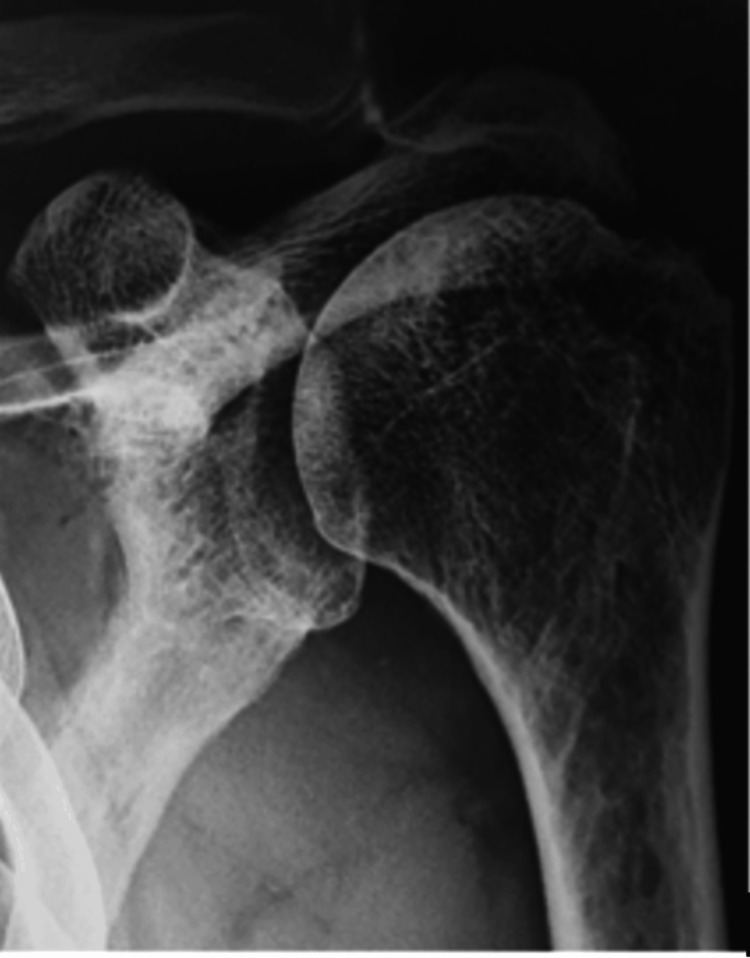
Anteroposterior radiograph of the left shoulder Superior migration of the humeral head is observed, with reduction of the acromiohumeral space to 6 mm, a finding consistent with a substantial rotator cuff tear

Clinically, he exhibited a full range of motion without scapular dyskinesis. The Jobe and Patte tests were positive, with no other relevant clinical findings. Radiographs revealed moderate humeral head migration with decreased subacromial space (Figure 1). 

**Figure 2:**
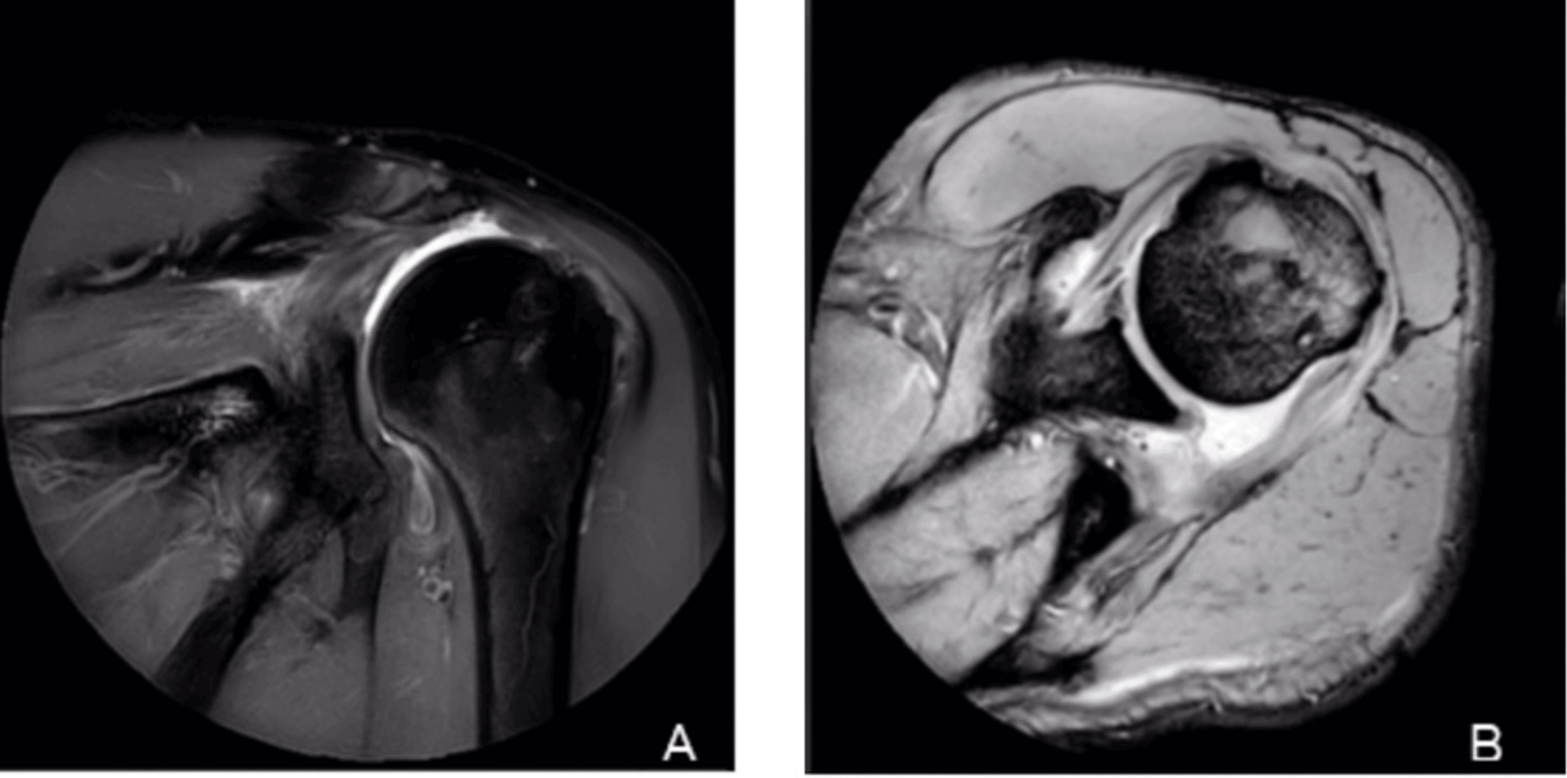
T2-weighted magnetic resonance imaging of the left shoulder (A) Sagittal view showing a retear of the posterior supraspinatus at the myotendinous junction with tendon retraction. (B) Axial view demonstrating extension of the tear to the infraspinatus

MRI showed a retear of the posterior portion of the supraspinatus at the myotendinous junction, extending to the infraspinatus. There was Goutallier grade III-IV fatty infiltration of both the infraspinatus and supraspinatus muscles (Figure 2). The indication for pursuing an arthroscopically assisted latissimus dorsi tendon transfer (LDTT) was revealed due to the imaging and clinical findings. 

Surgical technique 

The procedure was performed in three surgical stages, combining arthroscopic and mini-open approaches. The patient was placed in the beach-chair position, with the arm free at the side, and without traction. 

First Stage: Diagnostic Arthroscopy 

An exploratory arthroscopy was performed using the standard posterior, anterior, and lateral portals. The exploration identified a massive rotator cuff tear located at the myotendinous junction of the posterosuperior cuff, mainly involving the supraspinatus. The tear was found to be irreparable due to significant tendon retraction and poor-quality remaining tissue, thus contraindicating a new direct repair. Based on this finding, the decision was to perform an arthroscopically-assisted LDTT. 

Second Stage: Graft Harvesting (Mini-Open Approach) 

**Figure 3:**
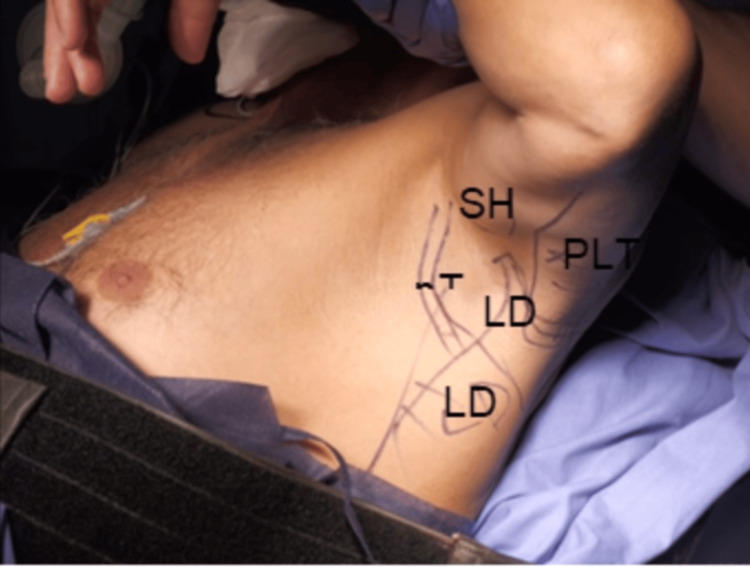
Mini-open axillary approach. Identification of anatomical structures LD: latissimus dorsi; LDt: latissimus dorsi tendon; SHB: short head of the biceps

The graft was obtained through a mini-open approach in the axillary region. With the patient still in the beach-chair position, the relevant anatomical structures were identified and marked: long head of the triceps, posterior deltoid, biceps brachii, LD, teres major, and the thoracodorsal neurovascular bundle (Figure 3).

**Figure 4:**
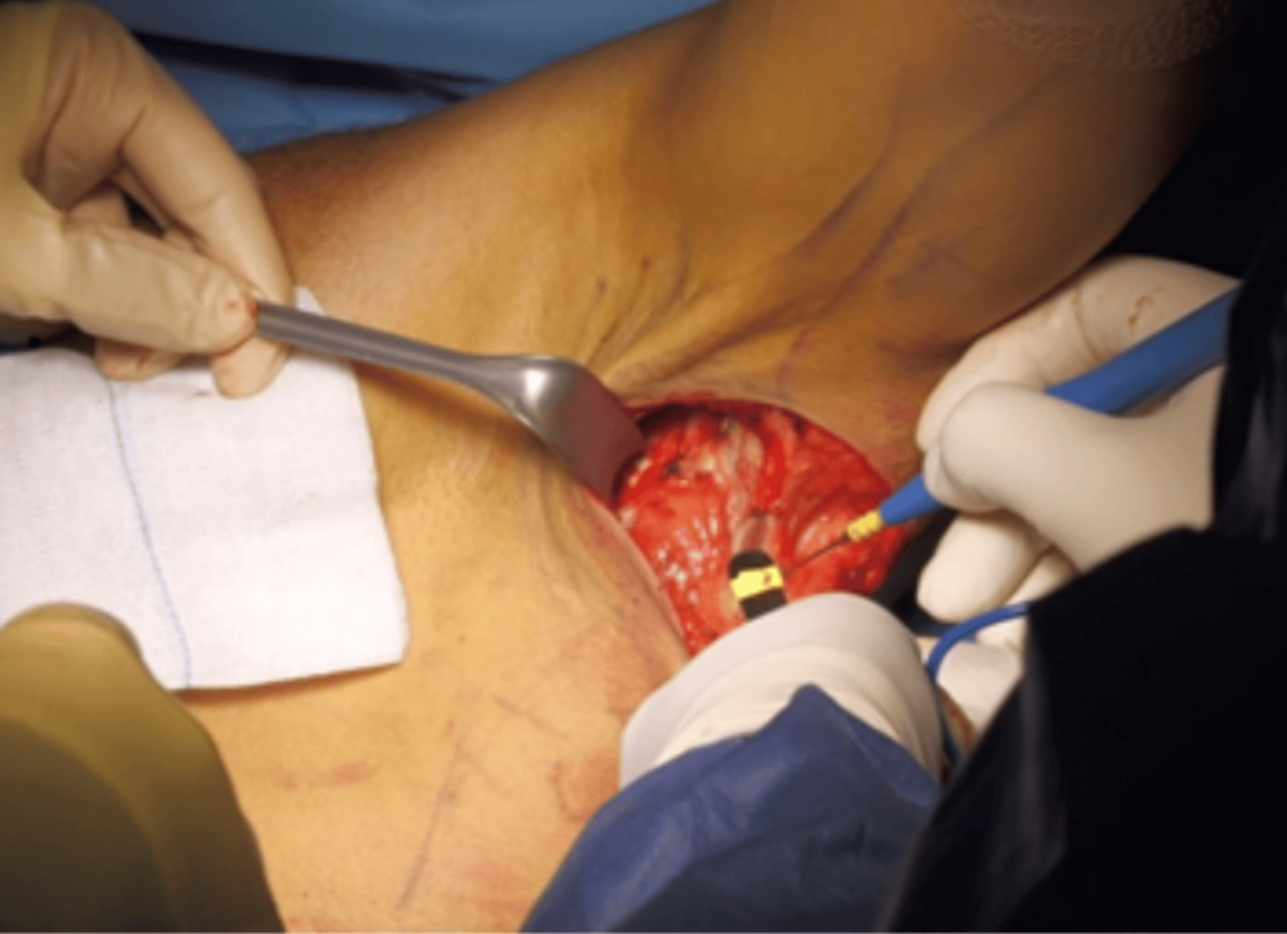
Incision. Perpendicular incision to the longitudinal axis of the latissimus dorsi tendon

A skin incision was made on the posterior aspect of the axilla, oriented perpendicular to the longitudinal axis of the LD tendon, which represented the most anterior structure of the posterior pillar of the axilla (Figure 4). 

**Figure 5:**
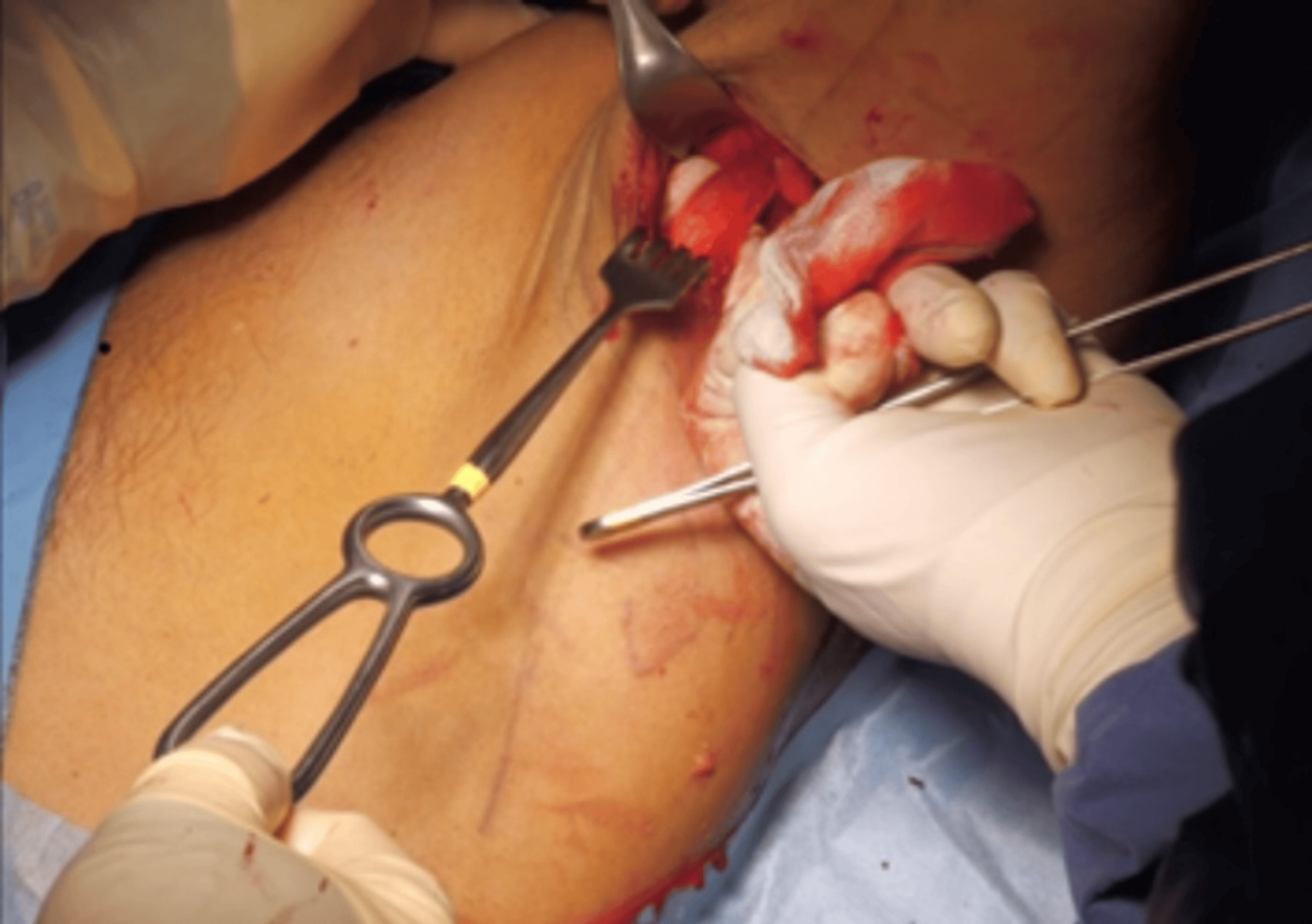
Dissection of the latissimus dorsi tendon

The LD tendon was carefully dissected and released from the teres major while preserving the integrity of the thoracodorsal nerve (Figure 5).

**Figure 6:**
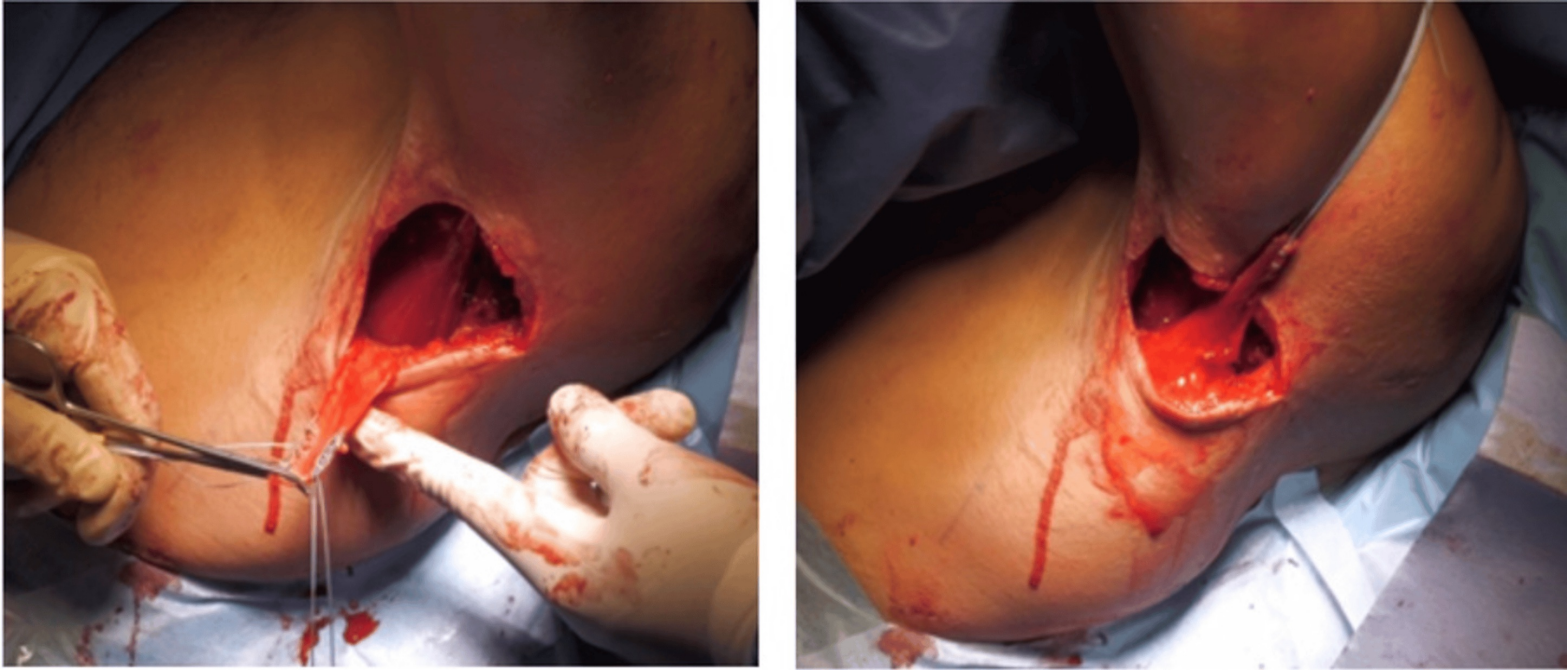
Harvesting and preparation of the tendon graft. Double row of high-strength sutures placed on the tendon end of the graft using the Krackow technique

After complete release, a 7 cm long graft was obtained. A soft-tissue plane was then developed between the triceps and teres minor to facilitate the passage of the graft medial to the axillary nerve toward the subdeltoid space. Graft preparation involved the placement of two high-strength sutures of different colors (one medial and one lateral) to facilitate manipulation and identification during arthroscopy (Figure 6).

The graft was passed through the posterior arthroscopic portal using a blunt instrument, after creating a space between several fascial planes: anteriorly, the deep plane to the posterior deltoid was identified and dissected; posteriorly, it was separated from the anterior border of the teres minor and the infraspinatus fascia; superiorly, it was kept below the trapezius and deltoid muscles. A posterior arthroscopic dissection was performed behind the infraspinatus, releasing the fascia and opening the space between the deltoid and infraspinatus until the axillary nerve was identified and left lateral to the blunt instrument used for the transfer. 

Third Stage: Arthroscopic Fixation of the Graft 

**Figure 7:**
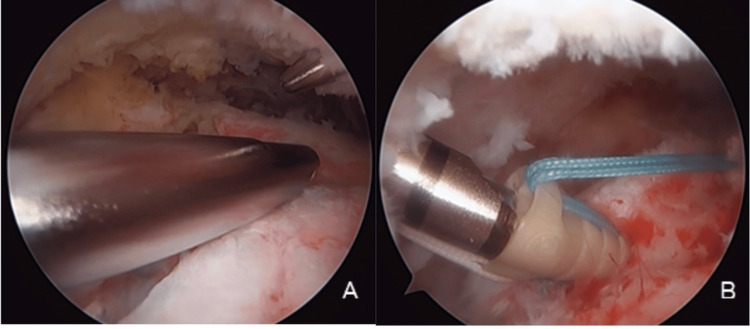
Footprint preparation along the entire extent of the supraspinatus and graft anchoring in the anterior region of the supraspinatus footprint

The footprint was prepared at the posterolateral aspect of the greater tuberosity. The graft was then tensioned and introduced into the subacromial space, covering the entire footprint up to the bicipital groove (Figure 7). 

**Figure 8:**
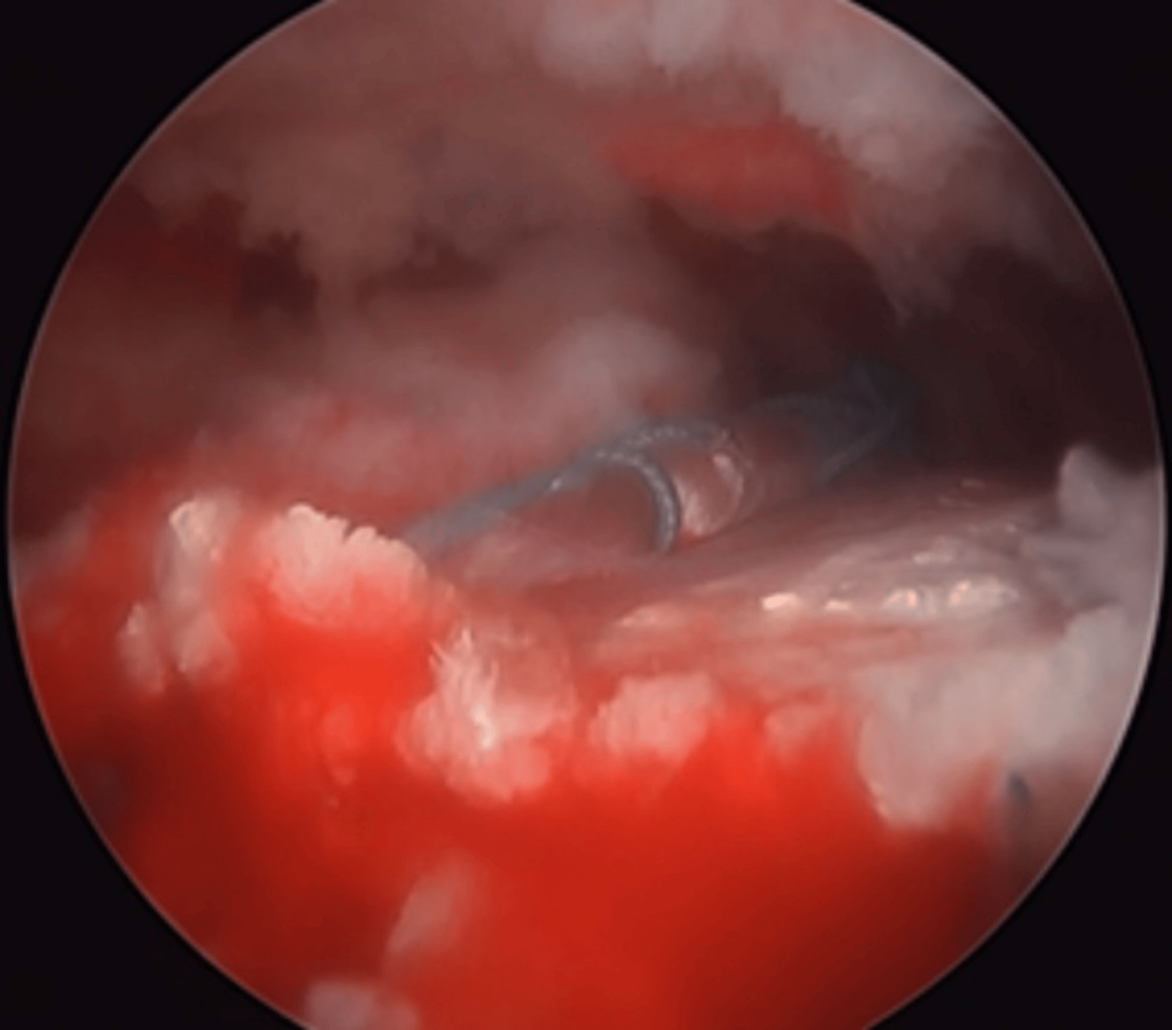
Tendon fixation using the suture bridge technique

Fixation was achieved using the transosseous-equivalent (suture bridge) technique, with two anterior anchors (ReelX®, Stryker, San José, USA) using nonabsorbable sutures and two lateral all-suture anchors. The ReelX anchors were progressively tensioned, allowing gradual reduction of the LD and providing adequate coverage and compression of the graft over the rotator cuff footprint, subsequently promoting optimal tendon-to-bone contact and biological integration (Figure 8). 

Postoperative evolution 

After surgery, the patient was immobilized in an Ultrasling® sling with a 15° abduction pillow for six weeks. Gentle pendulum exercises, passive external rotation, and passive forward flexion up to 80° were allowed. A structured rehabilitation protocol was followed, where during the first postoperative month, only pendulum and selected passive exercises were permitted, with no active motion. At five weeks, painless assisted passive mobilization was initiated, and gentle active movements were allowed after six weeks. 

**Figure 9:**
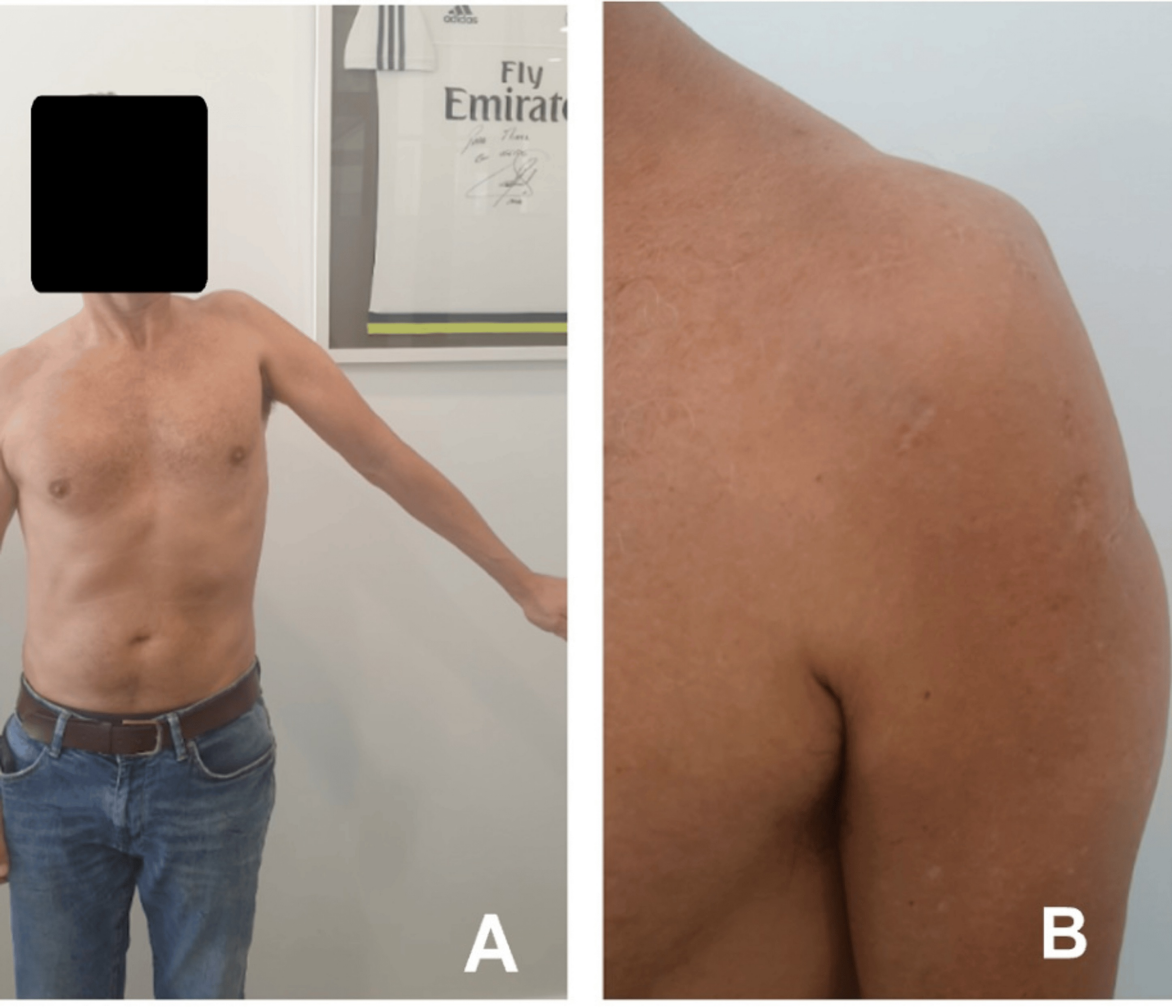
Sixteen-week postoperative follow-up (A) Shoulder abduction 45°; (B) muscle hypotrophy of the lateral deltoid portion

At seven weeks, the patient achieved assisted passive range of motion with forward flexion of 75° and abduction of 60°, but limited active motion (30° forward flexion and 30° abduction). By week 16, active forward flexion improved to 60° and abduction to 45° (Figure 9). However, atrophy of the lateral and posterior deltoid portions was evident (Figure 9), accompanied by hypoesthesia in the cutaneous territory of the axillary nerve and a positive “shield” sign, prompting specialized neurological evaluation. 

An electromyography (EMG) performed five months postoperatively revealed severe axillary nerve neuropraxia with signs of axonal degeneration. Based on this finding, surgical exploration versus conservative management was discussed. A watchful waiting strategy was chosen, with close follow-up and progressive functional rehabilitation. 

**Figure 10:**
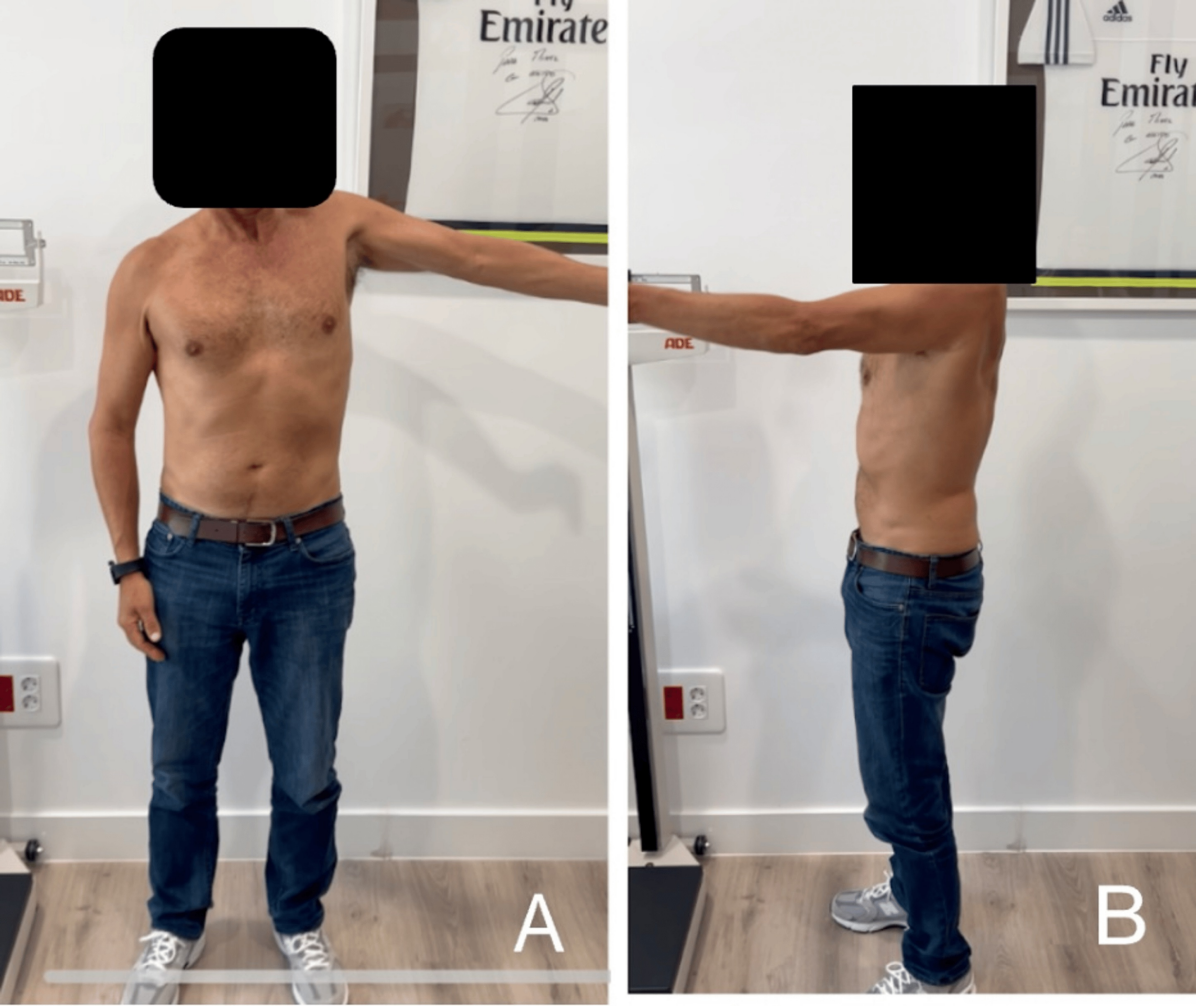
Ten-month postoperative follow-up (A) Abduction 85°; (B) flexion 90°

At six months, the patient showed progressive improvement, achieving active forward flexion of 75°, abduction of 60°, and external rotation of 40°, although deltoid atrophy persisted. At ten months, the functional range of motion improved to 90° forward flexion and 85° abduction (Figure 10), with a second EMG showing improvement in axillary nerve conduction, especially in the lateral and posterior deltoid portions. 

**Figure 11:**
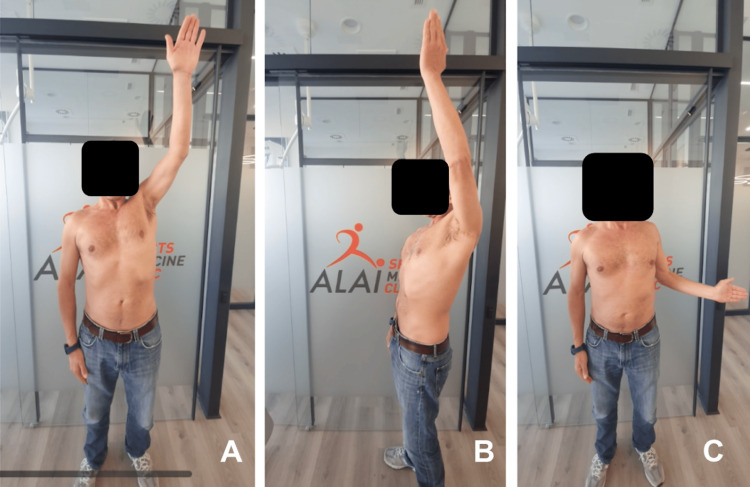
Fourteen-month postoperative follow-up (A) Forward flexion 170°; (B) forward flexion 170°; (C) external rotation 60°

At 14 months postoperatively, the patient achieved 170° of forward flexion, full external rotation, and complete recovery of deltoid muscle trophism without residual sensory deficits (Figure 11). 

He was discharged with satisfactory shoulder function and no objective neurological sequelae. Functional assessment using the Constant-Murley score yielded 88 points, indicating a highly satisfactory outcome. 

## Discussion

Retear of the rotator cuff is one of the main complications associated with this pathology, and its risk factors have been well-characterized. In the present case, the patient had previously undergone surgical repair and presented several risk factors such as age, tear size, and degree of fatty infiltration [9]. Moreover, a linear relationship has been described between the level of physical activity and the risk of injury. For example, athletes involved in “overhead” activities, as in this case, are more susceptible to risk of injury [9]. Another factor associated with a worse prognosis was the initial tear of the supraspinatus and infraspinatus at the myotendinous junction, which is technically more challenging to repair due to limited proximal tendon tissue available for suture fixation and retraction. The risk of retear in massive myotendinous junction tears exceeds 20% [10]. 

The main goal of treatment is to restore rotator cuff function while minimizing additional structural damage. This requires a comprehensive evaluation of the lesion characteristics, including patient age, symptoms, tendon tissue quality, and the general condition of the shoulder joint. In this case, the patient’s age and high functional activity level favored a surgical approach. However, due to the extent of the tendon damage, advanced fatty degeneration, and observed muscular atrophy, revision repair was ruled out from the beginning [10]. Similarly, superior capsular reconstruction was not considered due to loss of the shoulder’s rotational center, evidenced by superior migration of the humeral head on radiographs [11]. Consequently, an LD tendon transfer was chosen. 

Tendon transfer is primarily indicated in young, active patients with irreparable posterosuperior rotator cuff tears, for whom arthroplasty is not a viable option. This surgical technique´s purpose is to restore glenohumeral biomechanics, relieve pain, and prevent progression toward early degenerative arthropathy [3]. Two of the most widely recognized techniques are the LDTT described by Gerber et al. [5], and the lower trapezius transfer (LTT) proposed by Elhassan et al. [4]. Both techniques have demonstrated significant improvements in shoulder function and range of motion, with comparable clinical outcomes [12]. 

A critical criterion in selecting the appropriate transfer is the functional integrity of the subscapularis muscle. LDTT requires a competent subscapularis to maintain internal rotation and provide anterior stability, both essential for surgical success [1,3,5]. In the present case, this condition was met, which supported the selection of the LD transfer as a biomechanically favorable option. 

Conversely, although the LTT is more anatomically aligned with the force vector of the posterosuperior cuff, it is technically more demanding, frequently requires allograft augmentation, and is associated with a prolonged rehabilitation period [12]. Moreover, its use may be limited in cases with loss of the glenohumeral rotational center or advanced humeral head migration, as observed in the present case. Therefore, considering the preserved subscapularis, lower surgical complexity, absence of allograft requirement, and humeral head migration, LDTT represented the most suitable and effective alternative for this clinical scenario. 

Arthroscopy offers distinct advantages over open double-incision approaches, as it allows a detailed evaluation of the joint and subacromial space and facilitates the treatment of associated lesions. It also enables precise footprint preparation and anatomic graft placement with less soft tissue disruption [12]. Several studies have shown that arthroscopically assisted techniques are associated with less postoperative pain, lower rates of neurovascular complication, and faster functional recovery, all without compromising medium-term outcomes compared to open techniques [13]. 

Rehabilitation protocols must consider the progressive adaptation of the transferred muscle to its new function. Experimental animal studies have shown that while the muscle can rapidly increase its number of sarcomeres after elongation, tendon adaptation occurs more slowly, which may cause undesirable elongation if stressed prematurely [13]. Therefore, a conservative and gradual rehabilitation approach is justified to respect the graft’s biological adaptation. Allowing avoidance of early active loading and ensuring proper integration of the LD into its new traction vector [13]. Postoperative rehabilitation typically begins the day after surgery, starting with passive exercises during the first 4-6 weeks, followed by active mobilization after eight weeks, with gradual progression. 

The prevalence of complications after LDTT varies widely across studies, ranging from 7.3% to 18%, depending on the series reviewed. The most frequent complications involve wound issues such as infection, dehiscence, and hematoma [6,8], followed by tendon-related events such as failed integration or retear [14]. In the largest systematic review to date, an analysis of 421 patients with follow-ups of four to nine years reported that neurological complications were the least common. Only one case of axillary nerve neuropraxia was reported, with the radial, median, and ulnar nerves being more frequently affected [15]. 

Although neurological complications are among the least common in LD transfers [16], their consideration is crucial given the complex anatomy of the region and the proximity of critical neurovascular structures. The axillary nerve is at particular risk during two key stages: graft dissection and preparation, as well as, passage and fixation into the subacromial space. To minimize risk during graft harvesting, accurate anatomical identification is essential, and positioning the arm in flexion and internal rotation increases the distance between the axillary nerve and the LD tendon [17,18]. 

The second critical stage occurs during graft transfer into the subacromial space. Theoretically, if the graft is routed anterior to the triceps tendon, it may place the axillary nerve at risk of compression or injury, either during graft passage or subsequent muscle contraction. However, Petriccioli et al. reported safe passage of the LD tendon anterior to the triceps and either below or above the teres minor through the quadrilateral space, with no axillary nerve injuries in a series of 25 patients [19]. 

The arthroscopically assisted approach, as performed in this case, appears to offer the safest neurovascular profile. To further optimize safety, Petriccioli described a variant in which only the release of the LD muscle and tendon is performed via a mini-open approach [19]. This modification allows direct visualization of both the radial nerve during graft harvesting and the axillary nerve during subacromial passage, significantly reducing neurological risk. 

Neuropraxia typically presents with delayed recovery of motion, muscle weakness and atrophy, and sensory disturbances, as seen in our patient [20]. A significant traction applied to the tendon transfer with the ReelX anchor to cover the RC footprint was considered the most likely cause of the axillary nerve neuropraxia. 

The definitive diagnosis is made by EMG [20]. Although rare, neuropraxia is generally transient and shows spontaneous recovery within weeks to months, without the need for additional surgery [20]. Surgical intervention is considered if no improvement occurs within six months [20]. In our case, functional recovery began within this timeframe, confirmed by a follow-up EMG. 

The patient’s favorable neurological evolution, corroborated by nerve conduction studies, allowed continuation of rehabilitation without further interventions. Ultimately, the patient achieved satisfactory functional recovery, with a full range of motion, strength, muscle trophism, and complete sensory recovery of the axillary nerve. 

This case highlights the importance of a comprehensive evaluation for selecting the appropriate tendon transfer in irreparable rotator cuff tears, as well as the value of close follow-up and progressive rehabilitation protocols to ensure functional recovery, even in the presence of transient neurological complications.

## Conclusions

Axillary nerve neuropraxia is an uncommon complication in this type of surgical procedure. It is typically transient; however, timely diagnosis through neurophysiological studies is essential to ensure proper continuation of rehabilitation protocols and close follow-up to confirm spontaneous resolution. The favorable postoperative course in this case supports a conservative management approach for such postoperative neuropathies, emphasizing the importance of early clinical recognition, periodic monitoring, and understanding of the biological timeline of neural regeneration. 
